# Methicillin-Resistant *Staphylococcus aureus* ST398 in Swine Farm Personnel, Belgium

**DOI:** 10.3201/eid1507.080652

**Published:** 2009-07

**Authors:** Olivier Denis, Carl Suetens, Marie Hallin, Boudewijn Catry, Ilse Ramboer, Marc Dispas, Glenda Willems, Bart Gordts, Patrick Butaye, Marc J. Struelens

**Affiliations:** Université Libre de Bruxelles Hôpital Erasme, Brussels, Belgium (O. Denis, M. Hallin, M.J. Struelens); Scientific Institute of Public Health, Brussels (C. Suetens, B. Catry, I. Ramboer); Veterinary and Agrochemical Research Centre, Brussels (M. Dispas, G. Willems, P. Butaye); AZ Sint-Jan, Brugge, Belgium (B. Gordts); 1 Current affiliation: European Centre for Disease Prevention and Control, Stockholm, Sweden.

**Keywords:** MRSA, ST398, antimicrobial resistance, colonization, swine farm, MSSA, bacteria, staphylococci, Belgium, dispatch

## Abstract

We assessed methicillin-resistant *Staphylococcus aureus* (MRSA) in persons on 49 swine farms in Belgium. Surveys showed that 48 (37.8%) persons carried MRSA ST398 and 1 (0.8%) had concurrent skin infection. Risk factors for carriage were MRSA carriage by pigs, regular contact with pigs and companion animals, and use of protective clothing.

Prevalence of methicillin-resistant *Staphylococcus aureus* (MRSA) carriage has been high (>10%) among swine and exposed farmers and veterinarians (*1*,*2*). These MRSA strains are genetically unrelated to hospital- or community-acquired clones. They are resistant to digestion with *Sma*I and belong to ST398 (*1*). We assessed prevalence and characteristics of MRSA carriage and infection and associated risk factors for swine farm residents and workers in Belgium during 2007.

## The Study

We randomly selected 50 farms from the 7,500 farms in the pig farm national database of the Belgian Federal Agency for Food Safety. Sample size was based on an estimated 20% prevalence of MRSA colonization among farm workers and residents (*1*). The sample size was calculated to test the null hypothesis of prevalence <10% with a power of 80%. Participation was offered to all persons on the farm at the time of the visit, including farmers, co-workers, and household members. After giving written consent, participants were screened for MRSA carriage and interviewed. Simultaneously, on the same farms, a study of MRSA carriage in 30 randomly selected pigs per farm was conducted by veterinarians (*3*).

Samples from anterior nares and skin lesions on hands or face of human participants were placed into Stuart transport medium (Copan, Italy), inoculated within 24 h into 7.5% NaCl brain–heart infusion enrichment broth, and subcultured after 24 h onto Chromagar MRSA (bioMérieux, Marcy l’Etoile, France) and mannitol salt agar (Becton Dickinson, Heidelberg, Germany). *S. aureus* isolates were identified by coagulase test and PCR for 16S rRNA, *mecA,* and *nuc* genes (*4*).

Isolates were genotyped by pulsed-field gel electrophoresis after *Sma*I macrorestriction, *spa* sequence typing (http://spaserver.ridom.de), and determination of staphylococcal cassette chromosome *mec* (SCC*mec*) type and accessory gene regulator (*agr*) polymorphism (*4*,*5*). Four MRSA isolates were further analyzed by multilocus sequence typing (www.mlst.net). Multiplex PCR was used to test for Panton-Valentine leukocidin, toxic shock syndrome toxin 1, and exfoliatin A and B genes (*6*).

Antimicrobial drug susceptibility was tested by the Vitek2 system (bioMérieux). Multiplex PCR was used to test for resistance genes *tetK*, *tetM*, *aac*(6′)-Ie + *aph*(2′′), *ant*(4′)-Ia, *aph*(3′)-IIIa, *ermA,* and *ermC* (*7*–*9*).

Data were analyzed by using Stata 9.2 (Statacorp, College Station, TX, USA). We calculated prevalence of MRSA carriage in humans and 95% confidence intervals (CIs) by using cluster survey analysis. We performed risk factor analysis by using multiple logistic regression for cluster surveys, adjusting for clustering within farms. p values <0.05 were considered significant.

From April through July 2007, veterinarians investigated 50 swine farms. Of 1,500 pigs from 34 farms, 663 (44.2%) carried MRSA (*3*). On 49 of these 50 farms, 127 persons agreed to participate. Nasal (127) and wound (5) swabs showed that 48 (37.8%, 95% CI 25.6%–50.0%) participants carried MRSA and 22 (17%, 95% CI 10.7%–23.9%) carried methicillin-susceptible *S. aureus* (MSSA). One (2%) of the nasal MRSA carriers had a hand lesion infected with MRSA ST398 and treated it with topical antiseptic. Cultures from wounds on 4 other participants were negative for MRSA. Carriers of MRSA and MSSA were found on 25 (51.0%) and 15 (30.6%) farms, respectively. Prevalence of MRSA carriage was 50% for participants on farms with MRSA-colonized pigs versus 3% on farms without colonized pigs (relative risk 16.5, 95% CI 2.4–114.9, p <0.001).

Univariate analysis showed MRSA carriage to be associated with being a farmer or farm co-worker, being male, having regular contact with animals (including goats, sheep, dogs, or cats) and, paradoxically, wearing gloves and apron and reporting occasional or regular hand disinfection with an antimicrobial product. Multivariate analysis showed MRSA carriage to be independently associated with MRSA prevalence among pigs at the farm, being a farmer with regular pig contact, reporting regular contact with dogs and horses, and reporting use of protective clothing (apron, gloves, or mask) (Table 1).

**Table 1:** Risk factors for carriage of methicillin-resistant *Staphylococcus aureus* among 127 persons on 49 pig farms, Belgium, 2007*

Variable	No. carriers	No. noncarriers	aOR (95% CI)	p value
MRSA prevalence among pigs, %				
0	1	32	1	NA
1–49	15	17	50.7 (9.1–283.6)	<0.001
50–84	16	17	90.3 (12.3–664.1)	<0.001
>85	16	13	85.2 (14.5–501.8)	<0.001
Occupation, pig contact				
No pig contact	4	23	1	NA
Other, >1 time/week	9	16	2.7 (0.4–17.5)	0.543
Pig farmer, >1 time/week	35	40	14.4 (3.7–55.5)	<0.001
Contact >1/week with				
Dogs	16	4	19.8 (4.3–91.2)	<0.001
Horses	7	4	4.8 (1.6–14.2)	0.006
Use of any barrier precaution†	39	59	8.0 (1.8–36.3)	0.008

*Multivariate analysis. aOR, adjusted odds ratio; CI, confidence interval; NA, not applicable. †Protective clothing, e.g., apron, gloves, mask.

**Figure 1:**
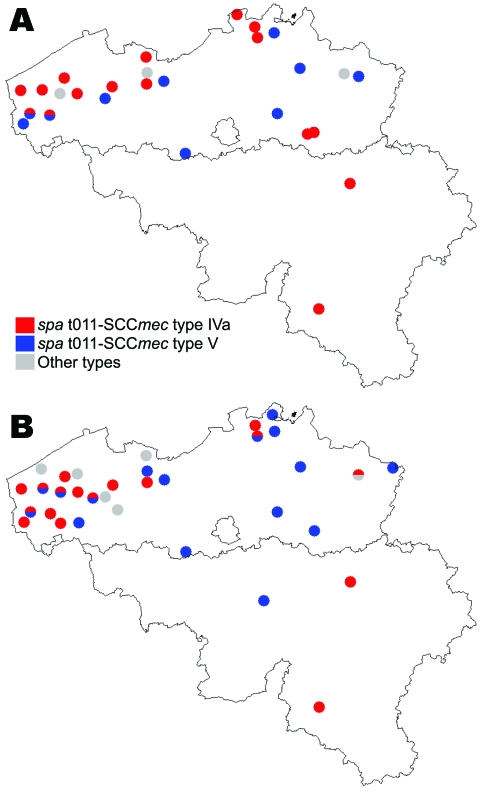
Distribution, by farms, of epidemic methicillin-resistant *Staphylococcus aureus* strains of *spa* type t011-SCC*mec* type IV, t011-SCC*mec* type V, and other types, Belgium, 2007. A) Farm residents and workers; B) Pigs. SCC, staphylococcal cassette chromosome.

**Figure 2:**
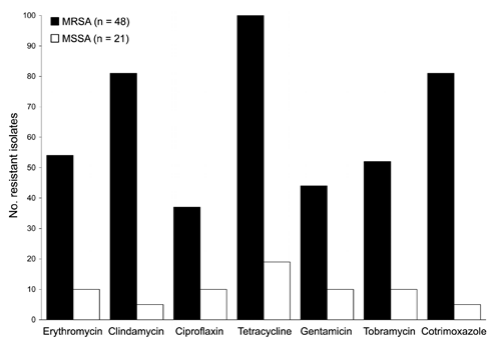
Antimicrobial drug susceptibility of *Staphylococcus aureus* strains recovered from pig farmers, Belgium, 2007. MRSA, methicillin-resistant *S. aureus*; MSSA, methicillin-susceptible *S. aureus.*

The 48 MRSA isolates were nontypeable by *Sma*I; some harbored SCC*mec* type IVa (n = 26), type V (n = 20), or were nontypeable (n = 2), and some exhibited 3 related *spa* types, t011 (n = 45), t034 (n = 2), and t567 (n = 1) (Table 2). Of the *spa* types, 4 representative strains belonged to ST398. Of the strains, 94% were classified into 2 genotypes, t011-SCC*mec* type IVa and t011-SCC*mec* type V, each found on 14 and 10 farms, respectively. In 8 of 11 farms with >2 MRSA carriers, all carriers harbored the same *spa*-SCC*mec* genotype. On 17 of 24 farms with MRSA colonization of humans and pigs, both groups carried the same genotype, suggesting animal-to-human transmission (Figure 1). Of MRSA isolates, 40 (83%) were resistant to tetracycline, cotrimoxazole, macrolides-lincosamides, aminoglycosides, and ciprofloxacin (Figure 2). Nearly all strains were susceptible to fusidic acid and mupirocin; all were susceptible to linezolid, rifampin, and glycopeptides. Resistance to aminoglycosides was conferred by the *aac*(6′)–*aph*(2′′) gene (n = 23) and the *ant*(4′) gene (n = 9). Resistance to macrolides-lincosamides was mainly mediated by *erm*C gene (n = 24). Tetracycline resistance was encoded by *tet*M and *tet*K genes in 48 (100%) and 23 (50%) isolates, respectively. Resistance profiles were related to clonal types (Table 2).

**Table 2:** Characteristics of 48 methicillin-resistant *Staphylococcus aureus* sequence type 398 isolates from persons on 49 pig farms, Belgium, 2007*

*spa* type	*spa* repeats	SCC*mec* type	No. isolates	No. farms					Methylase genes			Tetracycline resistance genes		Resistance phenotype (>50% isolates)
					AME genes									
					*aac*(6′)*–aph*(2′′)	*ant*(4′)	*aph*(3′)							
									*erm*A	*erm*C		*tet*K	*tet*M	
t011	08–16–02–25–34–24–25	IV	26	14	25	10	0		0	19		5	26	GEN, TOB, ERY, CLI, TET, SXT
		V	19	10	0	0	0		1	3		16	19	CIP, CLI, TET, SXT
t034	08–16–02–25–02–25–34–24–25	V	1	1	0	0	0		0	0		1	1	CIP, CLI, TET, SXT
		NT	1	1	0	1	0		0	0		1	1	TOB, TET
t567	08–02–25–24–25	NT	1	1	0	0	0		0	1		0	1	ERY, CLI, TET

*SCC, staphylococcal cassette chromosome; AME, aminoglycoside modifying enzyme; GEN, gentamicin; TOB, tobramycin; ERY, erythromycin; CLI, clindamycin; TET, tetracycline; SXT, cotrimoxazole; CIP, ciprofloxacin; NT, not typeable. All isolates were *agr* group 1.

MSSA isolates belonged to *spa* type t011 or t034 corresponding to ST398 (n = 3) and to 7 PGFE types and 12 *spa* types (n = 19). Genes encoding toxic shock syndrome toxin 1 (n = 4) and exfoliatin A (n = 3) were detected in 7 MSSA isolates. MSSA isolates were susceptible to all antimicrobial drugs except tetracycline (Figure 2).

## Conclusions

Human carriage of MRSA was associated with swine colonization with MRSA. Prevalence rate (38%) was higher than that for hospitalized patients or nursing home residents in Belgium (www.nsih.be/surv_mrsa/download_fr.asp). MRSA isolates from farmers belonged to closely related *spa* types corresponding to ST398, which are unrelated to hospital- and community-acquired strains but identical to strains from humans in contact with pigs in other European countries (*1*,*2*,*10*).

Despite the high prevalence of nasal MRSA, active MRSA skin infection was detected infrequently (<1%), within the range described in recent US-based studies (*11*). In a hospital in the Netherlands, a lower attack rate was found for MRSA ST398 than for other MRSA strains (*12*). However, invasive infections caused by MRSA ST398 have been reported, suggesting that this genotype is pathogenic for humans (*2*). In our study, MRSA strains did not harbor exotoxin.

Two MRSA genotypes were predominant. For 70% of farms with multiple MRSA carriers, all strains belonged to the same genotype, suggesting transmission within the farm. Although these strains have been shown to not spread easily in hospitals (*12*), outbreaks of MRSA ST398 in a residential care facility and a hospital probably originated from healthcare workers living on pig farms (*13*,*14*). In contrast with MRSA strains, MSSA isolates in our study showed diverse genotypes that frequently colonize human populations (*4*). MSSA isolates from 3 farmers belonged to the ST398 genotype, which is infrequently reported in humans except in pig farmers with contact with pigs (*4*).

Risk factors for MRSA ST398 carriage included regular contact with pigs but also with horses and dogs (*10*), suggesting that different animals could be MRSA ST398 reservoirs or vectors, at least on pig farms. Protective measures did not seem to reduce the risk of becoming colonized with MRSA; this lack of effectiveness has previously been observed for veterinarians (*15*). This apparent lack of protection should be further investigated to determine routes of transmission other than direct contact with pigs, including airborne transmission and contact with contaminated surfaces and companion animals.
